# Regional variation in cost of neonatal intensive care for extremely preterm infants

**DOI:** 10.1186/s12887-021-02600-8

**Published:** 2021-03-17

**Authors:** Asaph Rolnitsky, David Urbach, Sharon Unger, Chaim M. Bell

**Affiliations:** 1 Sunnybrook Health Sciences Centre, University of Toronto, 2075 Bayview Avenue, M4 wing NICU, Toronto, Ontario M4N3M5 Canada; 2 Surgery and Health Policy Management and Evaluation, University of Toronto, Women’s College Hospital, Toronto, Ontario Canada; 3 Paediatrics, University of Toronto, Mount Sinai Hospital, Toronto, Ontario Canada; 4 Medicine and Health Policy Management and Evaluation, Sinai Health System, University of Toronto, Toronto, Ontario Canada

## Abstract

**Background:**

Regional variation in cost of neonatal intensive care for extremely preterm infant is not documented. We sought to evaluate regional variation that may lead to benchmarking and cost saving.

**Methods:**

An analysis of a Canadian national costing data from the payor perspective. We included all liveborn 23–28-week preterm infants in 2011–2015. We calculated variation in costs between provinces using non-parametric tests and a generalized linear model to evaluate cost variation after adjustment for gestational age, survival, and length of stay.

**Results:**

We analysed 6932 infant records. The median total cost for all infants was $66,668 (Inter-Quartile Range (IQR): $4920–$125,551). Medians for the regions varied more than two-fold and ranged from $48,144 in Ontario to $122,526 in Saskatchewan. Median cost for infants who survived the first 3 days of life was $91,000 (IQR: $56,500–$188,757). Median daily cost for all infants was $1940 (IQR: $1518–$2619). Regional variation was significant after adjusting for survival more than 3 days, length of stay, gestational age, and year (pseudo-R^2^ = 0.9, *p* < 0.01). Applying the model on the second lowest-cost region to the rest of the regions resulted in a total savings of $71,768,361(95%CI: $65,527,634–$81,129,451) over the 5-year period ($14,353,672 annually), or over 11% savings for the total program cost of $643,837,303 over the study period.

**Conclusion:**

Costs of neonatal intensive care are high. There is large regional variation that persists after adjustment for length of stay and survival. Our results can be used for benchmarking and as a target for focused cost optimization, savings, and investment in healthcare.

## Table of contents summary

A national data analysis evaluated regional differences in cost of neonatal intensive care for preterm infants and the potential cost saving in benchmarking better performers.

## What is known on this subject

Neonatal intensive care for extremely premature infants (< 29 weeks) is prolonged and expensive. Regional variation has not been described in this population and can assist in cost reduction by learning from high performers.

## What this study adds

There is a wide regional variation in the remarkably high cost of neonatal intensive care that suggests a potential for benchmarking and focused cost savings.

## Background

Prematurity affects almost one in ten newborns [1], with 1 % of all newborns being extremely preterm (born before 29 weeks, or weighing less than 1500 grams [2, 3]). These fragile infants are often hospitalized for many weeks in a neonatal intensive care unit (NICU), requiring prolonged respiratory support, parenteral nutrition, and undergo many interventions such as ultrasonograms, surgeries, and blood tests. The complex care for this population involves multiple specialists in a level 3 (high acuity) NICU for several months. The intensive care provided is reflected in its high cost [4–6]. Extremely preterm infants accounted for some of the highest patient expenditures in hospitals [7, 8].

In recent years, support for infants born at 23 and 24 weeks gestational age, previously thought to be unviable, has become common in tertiary NICUs [2, 9, 10]. Indeed, most of these extremely preterm infants are resuscitated, with the majority surviving and being discharged home [2]. This has “pushed the envelope” for neonatal viability. Indeed, in many jurisdictions, it is standard practice to provide life support to newborns born at 23 or more weeks of gestation [9, 10].

Costs for providing care to this most vulnerable group have been uncertain [11–14]. Understanding these costs is important for health policy makers and planners in allocation decisions [15, 16]. As well, it has broad applicability since cost is considered a component of quality within the Institute of Healthcare Improvement’s (IHI) Quadruple Aim of Healthcare Quality [17]. Previous work with cost effectiveness analyses (CEAs) has estimated the cost-effectiveness of NICU care in various situations [18–26]. For example, neonatal resuscitation at 23 weeks had an estimated cost-utility of $15,134 to $22,256 per Quality-Adjusted Life Year (QALY) [19]. Variation in total cost can also affect the cost-effectiveness of the intervention.

As with all high-cost interventions, there is frequently wide variation in overall amounts. In this situation of extreme expense, documenting regional variation can help sites streamline processes and improve performance by learning from high performers. Thus, we sought to evaluate the cost and cost variation of care for these fragile preterm infants.

## Methods

### Data source

We used data from the Canadian Institute for Health Information (CIHI) database, a Canadian national agency responsible for the collection and analysis of health information. We received information on total cost of the neonatal stay from birth to discharge home or death, subcategorized by gestational age, province, and year. CIHI data is subject to quality checks, with ≥98% correlation with patient charts in multiple studies [27, 28]. Costing components are detailed in CIHI indicator library [29].

We included all newborn deliveries at 23–28 weeks gestational age, between January 1st, 2011 and December 31st, 2015. This represented years when 23-week infants began to be frequently supported in NICUs across Canada. There is usually a long delay in data availability as a result of extensive quality and audit checks precluding more current information.

We did not include Quebec as they do not submit data to CIHI. As well, the Canadian territories (Yukon, Northwest, and Nunavut) and the province of Prince Edward Island were excluded because of small numbers of deliveries and incomplete cost data. We also excluded stillbirths.

We used the province-submitted total cost for each patient for the complete neonatal hospital stay from birth to discharge home or death, including all hospital transfers. This excluded physician compensation. Which is not included in the database. Costing data is collected in the national database, CIHI, from the provinces using a standardized costing method [30]. This reflects the complete cost to the payor—the Ministries of Health—thereby providing a public perspective. Costing followed CIHI’s standardized approach [31–33]. Cost was adjusted to the published Canadian Healthcare Consumer Price Index [34] in 2011 Canadian dollars.

### Statistical analysis

Sunnybrook Hospital Research Ethics Board and CIHI approved the study protocol.

We calculated means, 95% confidence intervals [95%CI], medians, interquartile ranges [IQR] and standard deviations (SD) for each patient group. We compared groups using the Mann-Whitney-Wilcoxon test and Kruskal-Wallis test for non-normally distributed data. For variance, we used the Fligner-Killeen test for variance of multiple, non-normally distributed variables. For trends, we calculated the coefficient of determination (r^2^). We evaluated regional variation by adjusting for gestational age, length of stay, and year, using a multivariate analysis of cost. Length of stay was added to the multivariate analysis to correct for variation in hospitalization practices and discharge criteria variations. We calculated confidence intervals for each coefficient, pseudo-R^2^ and Akaike Information Criterion (AIC) to assess the model’s robustness. We repeated the model with cost data on infants who survived the first 3 days to accurately capture the cost impact of NICU stay, eliminating those who were too ill to survive or those who may have been withdrawn of life support. We also eliminated extreme outliers by calculating Cook’s D. Analyses were performed in R statistical language v4 and SPSS v21.

## Results

We analysed the costs for 6932 extremely preterm infants from 2011 to 2015 (Table 1). There were 5033 infants who survived more than 3 days. The absolute numbers of births for the 23–28-week age group was relatively constant year to year. The proportion of 23- and 24-week infants related to the total of 28 weeks and under was stable and ranged from 22.3–25.4% during the years of study (*p* = 0.5). Ontario accounted for 50.3% of all infant data, and Alberta, British Columbia, and Ontario together accounted for to 83% of the infants in all ages. For 23-week infants, Ontario accounted for 56% of the cohort. The proportion of 23-week infants was stable during the study years.

**Table 1:** Number of extremely premature infants admitted, length of Stay, Total cost and daily cost by gestational age and province, excludes Canadian provinces of Quebec and Prince Edward Island, and the Canadian territories

Province	Gestational Age (Weeks)	n	%n	Length of Stay (Days)					Cost (CAD)					Daily Cost (CAD)				
				Min.	Median	Mean	Max.	IQR	min.	Median	Mean	Max.	IQR	Min.	Median	Mean	Max.	IQR
**All**	23	699	10.08%	1	1	21.4	272	6	950	2294	45,978.8	743,360	23,329	633	2012	2622	146,939	1101
	24	933	13.46%	1	21	53.7	1011	103	645	55,290	112,767.5	1,577,166	220,515	645	2379	2627.1	84,852	1083
	25	1065	15.36%	1	55	59	415	94	1422	97,398	123,043	816,337	201,981	722	2186	2516	94,451	1116
	26	1227	17.70%	1	56	56.5	270	74	813	87,436	109,685	662,927	124,666	496	1943	2191.8	62,612	1161
	27	1366	19.71%	1	48	49.7	395	55	813	72,193	90,246	813,162	77,111	407	1789	2196.5	71,547	1056
	28	1642	23.69%	1	40	41	371	52	804	58,778	71,611.5	599,894	65,714	581	1653	1914.3	32,599	866
	< 26	2697	38.9%	1	17	47.4	1011	91	645	44,482	99,515.1	1,577,166	174,862	633	2179	2582	146,939	1143
	23–28	6932		1	41	48	1011	76	645	66,668	92,879	1,577,166	120,631	407	1940	2278.8	146,939	1103
**AB**	23	113	8.2%	1	2	35.1	171	72	2018	9847	94,980	743,360	192,864	1769	2763	4400	146,939	1513
	24	198	14.3%	1	35	53.5	164	102	2018	99,551	142,704	576,323	266,083	1125	2812	3123	7446	1246
	25	218	15.8%	1	60	57.8	234	81	2018	127,449	147,628	610,540	198,446	891	2621	3213.4	91,423	1011
	26	235	17.0%	1	60	55.8	159	62	1836	120,062	140,598	662,927	130,200	1066	2618	2734	8423	1334
	27	270	19.5%	1	48	47.1	141	45	2021	94,078	105,924	352,982	75,042	971	2182	2491.6	9659	1047
	28	349	25.2%	1	35	37.5	122	44	1836	71,281	83,126	453,104	76,878	944	2144	2392.2	4925	953
	< 26	529	38.3%	1	40	51.3	234	97	2018	102,602	134,539	743,360	241,644	891	2754	3433.2	146,939	1190
	23–28	1383		1	44	47.8	234	67	1836	93,151	117,008	743,360	129,602	891	2500	2867.8	146,939	1165
**BC**	23	76	8.5%	1	1	25	182	7	1018	2165	52,508	582,267	29,772	1017	1780	2161	4913	729
	24	113	12.6%	1	68	69.1	386	112	1537	111,392	137,693	788,913	228,354	1081	2024	2223	3943	936
	25	124	13.8%	1	48	59.2	415	99	1546	93,312	115,980	816,337	195,154	947	2118	2261	4146	868
	26	164	18.3%	1	70	63.2	212	82	1251	93,184	111,824	458,446	146,223	778	1851	1910.4	3979	926
	27	182	20.3%	1	64	61.3	395	46	1527	77,792	98,460	813,162	61,571	612	1546	1736.2	4025	897
	28	237	26.5%	1	47	48.4	371	49	1336	64,509	74,783	599,894	55,101	582	1463	1667.5	3593	747
	< 26	313	34.9%	1	29	54.5	415	100	1018	63,487	108,407	816,337	198,000	947	2024	2223.2	4913	909
	23–28	896		1	52	55.9	415	82	1018	76,735	98,118	816,337	129,361	582	1777	1920	4913	893
**MB**	23	36	10.2%	1	1	11.8	137	0	1023	1782	22,376	271,603	657	1023	1750	1930	4819	140
	24	38	10.8%	1	16	67	235	120	1652	52,766	138,972	565,165	244,226	1024	2224	2452	5265	1148
	25	60	17.0%	1	92	74.3	206	115	1652	117,909	149,080	507,058	231,558	893	2292	2483.4	5801	1460
	26	70	19.9%	1	92	75	175	97	1496	113,850	140,835	297,071	182,751	784	2023	2205.6	5735	1056
	27	64	18.2%	1	75	63.8	130	64	885	98,092	112,683.7	291,343	158,379	760	1964	2690.1	50,717	1265
	28	84	23.9%	1	59	58.3	179	28	1585	80,829	96,258	305,679	18,603	740	1530	1806.7	5361	773
	< 26	134	38.1%	1	12	55.4	235	112	1022	28,122	112,174	565,165	239,201	893	1995	2325.9	5801	1058
	23–28	352		1	63	61.5	235	97	885	84,423	114,168.1	565,165	204,335	740	1862	2244.3	50,717	1058
**NB**	23	9	6.52%	1	1	1.8	8	0	1515	1608	5703	35,324	1014	1515	1608	2269	4415	1014
	24	16	11.59%	1	14	22.3	110	16	1860	29,330	49,574	237,209	34,149	1439	2348	2399	3634	484
	25	8	5.80%	2	29	39	108	62	7203	57,356	72,741	202,599	104,119	1489	2289	2625	4489	1472
	26	26	18.84%	1	53	49.7	101	65	1453	73,990	106,394	223,810	154,912	664	2428	2306.2	4095	1217
	27	34	24.64%	1	68	57	106	38	1596	83,932	89,968	237,209	48,127	628	1494	1914	13,178	659
	28	45	32.61%	1	57	50.9	84	24	1480	74,260	76,349	211,229	34,141	631	1396	1636	3638	865
	< 26	33	23.9%	1	3	20.8	110	16	1515	12,951	43,226	237,209	42,222	1439	2257	2418	4489	921
	23–28	138		1	54	45	110	55	1453	72,956	77,444	237,209	55,951	628	1661	2018	13,178	1242
**NL**	23	17	14.29%	1	1	13.5	185	6	1818	1907	24,678	278,211	16,828	1504	1907	2382	5112	843
	24	15	12.61%	1	113	98.4	426	134	1668	236,013	188,786	895,475	257,035	1356	2041	2272	3853	810
	25	19	15.97%	1	125	109.2	248	33	2758	265,420	250,195	533,730	116,870	1148	2230	2568	4937	680
	26	17	14.29%	1	88	88.8	260	97	1473	197,098	188,131	661,937	262,666	984	2119	2125	3205	1240
	27	22	18.49%	1	81	82.5	260	32	1668	219,382	179,956	608,293	161,148	1131	2079	2203	4072	1263
	28	29	24.37%	1	68	62.7	137	21	1659	107,445	128,643	265,420	120,545	756	1937	2179.9	3981	1279
	< 26	51	42.9%	1	23	74.1	426	127	1668	98,764	156,961	895,475	263,124	1148	2092	2419	5112	916
	23–28	119		1	69	75	426	110	1473	107,446	158,764	895,475	244,145	756	2079	2278.8	5112	1100
**NS**	23	17	7.5%	1	7	52.2	199	95	1584	68,400	98,682	289,819	172,406	1211	1778	6046	70,843	464
	24	39	17.3%	1	106	117.6	1011	123	1626	230,891	220,282	1,577,166	250,440	696	2384	2307	4091	955
	25	27	11.9%	1	100	95.2	346	66	1765	205,783	181,488	607,373	149,881	935	2021	2123.8	4236	762
	26	35	15.5%	1	87	91.6	220	36	1503	202,290	174,881	475,022	147,518	753	1961	2024.5	3445	982
	27	49	21.7%	1	78	78.4	193	33	1599	102,039	137,757	436,276	118,970	703	1599	1786.9	3788	795
	28	59	26.1%	1	66	66	181	25	2093	89,249	112,667	300,851	37,030	930	1517	1748.8	4086	840
	< 26	83	36.7%	1	99	96.9	1011	126	1584	175,442	182,756	1,577,166	249,140	696	2077	3013.1	70,843	933
	23–28	226		1	77	84	1011	53	1503	103,515	153,482	1,577,166	155,619	696	1778	2264.1	70,843	988
**ON**	23	395	11.3%	1	1	17.9	272	2	950	2086	33,727.4	523,396	7079	633	1693	2120	13,449	1079
	24	464	13.3%	1	13	44.4	343	90	645	35,399	82,091.4	636,658	144,203	645	2088	2539.8	84,852	977
	25	552	15.8%	1	42	52.5	364	84	1422	78,565	95,991	743,222	136,611	722	1956	2277	94,451	909
	26	616	17.6%	1	49	49	270	70	813	71,306	82,063	542,585	87,721	496	1713	2007.5	62,612	922
	27	683	19.6%	1	38	40.8	218	55	813	54,472	64,745	456,931	63,708	407	1582	2149.2	71,547	807
	28	781	22.4%	1	29	33.6	165	45	804	40,524	50,655.8	454,455	58,179	581	1494	1737.5	32,599	632
	< 26	1411	40.4%	1	9	40.2	364	75	645	27,614	73,989.9	743,222	108,605	633	1957	2319	94,451	1064
	23–28	3491		1	29	40.4	364	65	645	48,144	68,385.5	743,222	87,812	407	1712	2100.9	94,451	962
**SK**	23	36	11.0%	1	1	12.5	118	0	1876	2098	31,655	302,674	1845	1876	2098	2805	16,285	441
	24	50	15.3%	1	12	43.1	151	100	1728	54,140	116,202	355,019	276,994	795	2746	2945.8	5958	1446
	25	57	17.4%	1	92	78.8	214	85	2048	273,698	215,947	646,856	197,285	812	2906	2902.2	5434	865
	26	64	19.6%	1	78	68.1	193	88	1942	160,914	167,326	368,115	229,716	1004	2722	2743	5263	1187
	27	62	19.0%	1	79	73	136	33	2699	233,511	186,384	334,666	166,386	904	2736	2750.5	5352	1565
	28	58	17.7%	1	44	56.6	204	102	1630	116,113	161,897	331,300	162,243	1108	2673	2836	13,886	2160
	< 26	143	43.7%	1	18	49.6	214	102	1728	62,846	134,676	646,856	283,360	795	2657	2893	16,285	1257
	23–28	327		1	69	59.7	214	95	1630	122,526	155,698	646,856	265,411	795	2695	2826.5	16,285	1462

### Length of stay

The median length of stay (LOS) was 41 days (IQR: 1–77). Ontario had the lowest median LOS (29 days, IQR: 1–66) and Nova Scotia had the highest median LOS of 77 days (IQR: 53–106). (Table 1) For infants who survived more than 3 days, the median LOS was 61 days (IQR: 34–90) and ranged from 51 days (IQR: 27–82) in Ontario to 88 days (IQR: 64–126) in Newfoundland.

### Cost

**Fig. 1:**
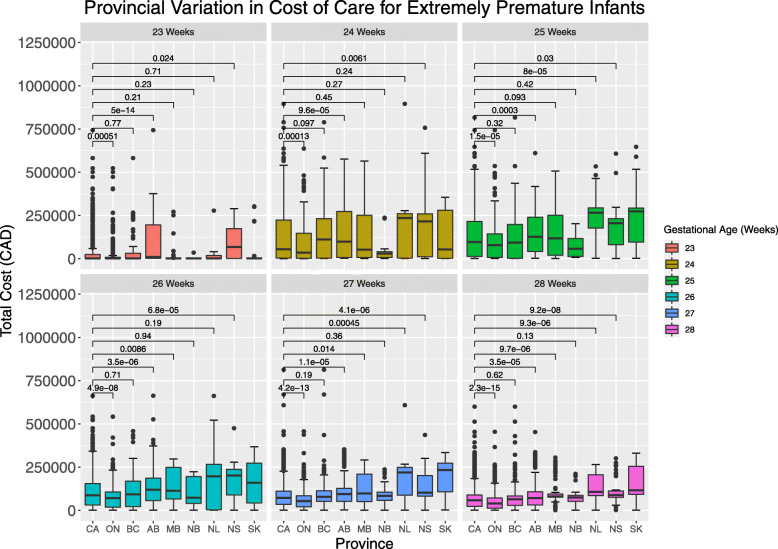
Cost variation between Canadian provinces, by gestational age. CA – Combined, included provinces, AB- Alberta, BC- British Columbia, MB- Manitoba, NB- New Brunswick, NL- Newfoundland and Labrador, NS- Nova Scotia, ON- Ontario, SK- Saskatchewan. *p* values for Wilcoxon test

The median total cost was $66,668 (IQR: $4920–$125,551). This ranged from $48,144 in Ontario (IQR: $2807–$90,619) to $122,526 in Saskatchewan (IQR: $8288–$273,699). The lowest costing for the entire regional cohort was in Ontario, with median cost of $48,144 (IQR: $2807–$90,619), and the second lowest was in New Brunswick, with median cost of $72,956 (IQR: $33,265–$89,216). Figure 1 demonstrates the regional variation in cost for the entire cohort by gestational age. For infants who survived more than 3 days, the median total cost was $91,137 (IQR: $56,596–$188,757). The median daily cost was $1940 (IQR: $1515–$2619) and ranged from $1661 in New Brunswick (IQR: $1325–$2567) to $2696 in Saskatchewan (IQR: $1958–$3420). The median daily cost for infants who survived more than 3 days was $1805 (IQR: $1392–$2419) and ranged from $1567 in New Brunswick (IQR: $1252–$2325) to $2764 in Saskatchewan (IQR: $1931–$3436). There was a small increase in the median total cost over the years of the study (r^2^ = 0.043 *p* < 0.001).

**Fig. 2:**
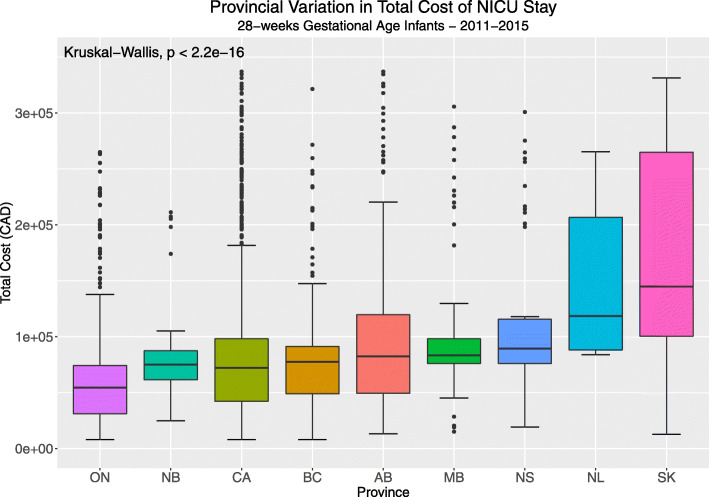
Regional variation in 28-week infants who survived the first 3 days of life, representing a relatively more mature and more stable preterm populations in our cohort. Kruskal-Wallis test for variance. CA – Combined, included provinces, AB- Alberta, BC- British Columbia, MB- Manitoba, NB- New Brunswick, NL- Newfoundland and Labrador, NS- Nova Scotia, ON- Ontario, SK- Saskatchewan

There was wide variation between regions even within similar age groups (Fig. 1). For example, median total costs for 25-week infants in Saskatchewan were as high as $273,698 while in Ontario the median was $78,565, and in New Brunswick it was $57,356, a 4.8-fold difference. We examined for regional cost variation for infants born at 28-week gestation (Fig. 2), a typically more stable population, with fewer complications of NICU stay. The median costs in Ontario were $40,524, in Manitoba they were $80,829, and in Saskatchewan they were $116,113, a 2.9-fold difference. There was wide regional variation in cost for every gestational age when compared to the entire cohort. The variation in costs of hospitalization between the regions for each age group were significant (*p* < 0.001). In a multivariate analysis using a generalized model, fitted to its Gamma distribution, and after elimination of extreme outliers, we demonstrated a persistent regional variation in cost of care after adjustment for length of stay, survival more than 3 days, gestational age, and year of study (*n* = 6890). For example, for 28-week infants, the adjusted variation was up to 1.87-fold in cost. This model was robust, demonstrated by a pseudo-R^2^ = 0.93, *p* < 0.001.

Using the model to estimate potential cost savings, we applied the lowest cost to the other regions in the cohort. The total cost saving calculated was $87,801,982 (95%CI: $95,783,981–$83,810,983) over the 5 years, representing 13.6% saving of the total budget of $643,837,303 over the same timeframe, or $17,560,396 annually.

For a more achievable benchmark [35],we applied the second-lowest cost region to the other regions in the cohort. This resulted in a total cost savings of $71,768,361 (95%CI: $65,527,634–$81,129,451) over the 5-year period. This represented 11.15% of the total budget of, or $14,353,672 annually.

## Discussion

We evaluated all extremely premature infants born in Canada from 2011 through 2015. We demonstrated high overall cost for premature infants and their complications. There was up to 8-fold regional variation in cost. The effects persisted even after adjustment for differences in survival, gestational age, length of stay, and year of birth. We found that overall, the median cost of care was $66,668 and for infants who survived more than 3 days median cost was $91,000. This did not change significantly over the study period. We also found that the median length of stay for the entire cohort was 41 days and did not change over time. Moreover, we found that significant savings could be achieved with benchmarking to lower cost regions. In a recent cost evaluation study, Rios et al. [36] reported the cost of tertiary NICU care using a predictive model, estimating the cost of the age group of < 29 week infants at $100,423 (IQR: $56,800–$159,358) and a mean daily cost of $1964. Our study differed in focusing on regional differences and the inclusion of the different age groups and stay at different level of hospital units.

Our study has several strengths. First, we used a reliable, quality-standardized, national-level dataset that includes cost and gestational age. Second, our study reflects data from time periods when infants born at 23 weeks gestation began to be routinely supported. Third, our findings follow the patient care pathway in the complete hospitalization from birth to discharge home or death. This includes hospital transfers to higher and lower acuity sites, thus providing the cost of care for the infant prolonged stay, at the provincial level, from the payor perspective. Fourth, our cost modelling shows robust, significant variation after adjustment for several variables.

Healthcare spending in Canada is determined regionally, where each province is responsible for most of its own healthcare services [37]. The coverage and costing are influenced by local healthcare policies in the context of local economies, and by differences in clinical practices, as well as medical decisions. Notably, regional differences in healthcare costs were demonstrated previously in other areas of healthcare [18–23] but not in NICU patients.

International reports through the World Health Organization (WHO) and the Organization for Economic Cooperation and Development (OECD) have compared national outcomes and financial performance in healthcare for many years. Regional variation has previously been reported in various healthcare expenditures [38–41] at the national level, both in per capita calculations and in relationship to GDP. National comparisons are fraught with difficulties in comparing like elements. In contrast, regional comparisons can often be more standardized. Indeed, regional cost differences have been demonstrated in cancer care [38], cochlear implants [42], tuberculosis care [43], and long-term care [44]. The latter, for example, demonstrated 5-fold variation in regional cost in the same country [44]. Quantifying this variation within a country is important for the regional policymakers to allocate resources, and for policymakers in other countries to compare and benchmark their results and variation. This variation is sometimes reflective of local policies and costing mechanisms. Our data differ because of the consistency in the costing and outcome methods. We found that the variation persisted regardless of the gestational age. Indeed, the variation in median total costs was striking even after rigorous adjustments. For example, median costs for infants born at 28 weeks gestation, a more stable population in this cohort, varied 2.9-fold between the regions. These differences persisted in the multivariate model, supporting the notion that regional variation contributed significantly to the cost of care. Examining the costs for 28-week infants is highly illustrative because their survival rate is close to 100%, and they would complete their stay to discharge. Indeed, their course is typically less complicated [2, 45] and expected to be less expensive. Therefore, regional practices and their inherent costs are more explanatory of the variation in their cost of care.

There are several potential causes for cost variation. Previously listed [46] drivers of healthcare cost are population complexity, physician billing, inflation, pharmaceuticals, materials, remunerations and administrative costs. Some have noted [35] that acuity and complexity can drive these cost differences. However, less is known about cost differences between jurisdictions when comparing the same condition with similar acuity. While there are demonstrable variations in specific cost components between regions, we currently cannot determine the specific causes, or subcategories, of the differences in our data [47]. This is well demonstrated in the fact that one province (SK) had higher median cost while having another had a relatively shorter length of stay (NFL). The differences may stem from local hospital costs, medication and procedural practices, and expensive interventions such as ventilation and parenteral nutrition. The variation in these practices are reflected in national level reports [2] but have not been translated to costs.

Our study has several limitations. First, we excluded some jurisdictions from the analysis due to availability of or quality of data. Nevertheless, we include over 70% of the national population. Additional data may only add to the observed variation. Second, as in many studies, our findings rely on coding accuracy and consistency of administrative data. However, the standardized approach to cost calculation that has been applied to acute care hospitals across Canada in CIHI methodologies [27, 28] was demonstrated to be highly accurate. This enables the calculation of accumulated cost of hospital stay of a preterm infant from birth, through hospital units or transfers, to discharge or demise. Third, our analyses considered only hospital costs from the birth to discharge home or death. It did not include health services in later life that many of these infants, who suffer from complications related to preterm birth, will require. While this may lead to an underestimate of costs, our focus was on the costing of entire hospital stay, thereby better reflecting the local policies. Fourth, the cost of care did not adjust for clinical outcomes or adverse events. These important aspects need to be included within an in-depth comparison of programs, which should be considered in future work. Fifth, we were unable to adjust for clinical practice differences (such as particular procedures, ventilation modes, staffing, or nutrition). This could assist in calculation of cost avoidance due to local systemic contributors to costing. Confidentiality agreements or data limitations prevented us from performing this type of analysis. Sixth, physician compensations are not included in this analysis since this is not reported to CIHI as part of the cost of care calculation. Although this puts an underestimation to the societal cost, this emphasizes even more the high cost in preterm care. Finally, we report cost of hospital stay without ethical consideration regarding quality of life, and without performing a formal cost-effectiveness or a cost-utility analysis. Indeed, ethics in the costs of medical care have been considered in other policy relevant work [14, 18–23, 48–50].

## Consclusions

We found extensive regional cost variation for extremely preterm infants. The findings persisted after adjusting for several predictive factors. These results demonstrate that there is much room for cost reduction and standardization in support of cost reduction, one of the quadruple aims of healthcare quality improvement [51]. Reducing large cost variation through standardization can lead to cost savings [52, 53]. Our findings may be useful to policymakers for planning and resource allocation decisions. Moreover, small cost differences can be amplified over large patient cohorts. In our study, even a small cost variation of 3% translated to large total differences of $2786 per patient and $19,315,117 in total. These were further magnified when potentially achievable amounts for lower cost regions were applied broadly and over several years [54]. Decreasing such variation can help centres and regions decrease their cost while maintaining excellent care. In time, this will allow for channelling the savings towards further investments and innovations to improve the care of these fragile infants.

## Data Availability

The datasets generated and/or analysed during the current study are not publicly available due to data sharing agreements but are available from the corresponding author on reasonable request.

## Abbreviations

AB: Alberta
BC: British Columbia
MB: Manitoba
NB: New Brunswick
NL: Newfoundland and Labrador
NS: Nova Scotia
ON: Ontario
SK: Saskatchewan
CAD: Canadian Dollar
GA: Gestational age
NICU: Neonatal intensive care unit
